# Effect of waste mango silage on the *in vitro* gas production, *in situ* digestibility, intake, apparent digestibility, and ruminal characteristics in calf diets

**DOI:** 10.14202/vetworld.2023.421-430

**Published:** 2023-03-15

**Authors:** Ulises Remo Cañaveral-Martínez, Paulino Sánchez-Santillán, Nicolás Torres-Salado, David Hernández-Sánchez, Jerónimo Herrera-Pérez, Marco Antonio Ayala-Monter

**Affiliations:** 1 Department of Animal Nutrition, Master in Bovine Production in the Tropics, School of Veterinary Medicine and Zootechnics No. 2 of the Autonomous University of Guerrero, Cuajinicuilapa, Guerrero, 41940, México; 2 Post Graduate Program of Livestock, Postgraduate College, Montecillos, Estado de México, 56230, México

**Keywords:** alternative feed, alternative feeding, cattle, silage, tropics

## Abstract

**Background and Aim:**

Mexico is the fifth largest producer of mangoes in the world. For the conservation of agro-industrial waste and crop residues, the ensiling technique has shown good results. This study aimed to evaluate the effect of increasing the level of mango silage (86% waste mango and 14% pangola grass hay) in calf diets on *in vitro* gas production, *in situ* digestibility, intake, apparent digestibility, and ruminal characteristics.

**Materials and Methods:**

The diets contained 0 (T0), 30 (T1), 45 (T2), and 60% (T3) mango silage. The partial (24, 48, and 72 h) and cumulative (72 h) biogas, CH_4_ production, and degradation were determined in the *in vitro* evaluation. *In situ* digestibility and estimators of fermentation kinetics of dry matter (DM) and organic matter (OM) were determined. Intake, apparent nutrient digestibility, and rumen parameters of calves (200 kg) were evaluated in a 4 × 4 Latin square design. Response to increased mango silage was calculated by linear and quadratic orthogonal contrasts.

**Results:**

*In vitro* partial and cumulative biogas production decreased linearly (p < 0.05), and the partial and cumulative CH_4_ production did not show linear or quadratic contrast (p > 0.05); *in vitro* DM degradation, *in vitr*o neutral detergent fiber degradation, and *in vitr*o acid detergent fiber degradation showed a linear increase (p < 0.05). *In sit*u dry matter digestibility (DMDis), *in situ* organic matter digestibility (OMDis), b, a + b, c, and effective digestibility (ED) of DMDis, a, a + b, c, and ED of OMDis increased linearly (p < 0.05). Dry matter intake, OM intake, and crude protein intake showed a linear increase (p < 0.05); NDF intake and ADF intake presented a quadratic behavior (p < 0.05). Apparent digestibility of DM, OM, CP, and hemicellulose, pH, N-NH_3_, total bacterial count, acetate, propionate, butyrate, volatile fatty acids, acetate: propionate ratio, cellulolytic bacteria, and protozoa did not present a linear or quadratic orthogonal effect (p > 0.05).

**Conclusion:**

The *in vitro*, *in situ*, and *in vivo* variables demonstrated that up to 60% mango silage can be used for the intensive fattening of calves in confinement.

## Introduction

Mexico is the fifth largest producer of mangoes (*Magnifica indica* L.) in the world, and the state of Guerrero is the main producer at the national level. For each mango that is produced, 28%–38% is peel and seed, which are considered to be waste products, in addition to harvest residues and agro-industrial waste [1]. These wastes form acidic effluents that contaminate the soil and water. The ensiling technique has shown good results for the conservation of agro-industrial wastes and crop residues [2].

The use of mango residues in silages and their utilization in ruminant feed have been evaluated in Mexico [3] and other countries [4, 5]. Using these residues to prepare silage is an alternative feed for ruminants in the tropics [3, 6].

This study aimed to evaluate the effect of using different dietary levels of waste mango silage on the *in* *vitro* gas production, *in situ* digestibility, intake, apparent nutrient digestibility, and ruminal characteristics in calves.

## Materials and Methods

### Ethical approval

The study was approved (No. 1/2019) by Animal Ethics Committee, the School of Veterinary Medicine and Zootechnics No. 2 of the Autonomous University of Guerrero, Mexico. The calves’ care and management procedures were conducted according to the guidelines established by the Animal Research Reporting of *In vivo* Experiments [7].

### Study period and location

The study was conducted from May 2020 to March 2021 in the facilities of the Zootechnical Posta and Animal Nutrition Laboratory of the School of Veterinary Medicine and Zootechnics No. 2 of the Autonomous University of Guerrero.

### Ensilage processing

Mango waste was collected from orchards located in Cuajinicuilapa, Guerrero, Mexico. Pangola grass (*Digitaria decumbens* L.) hay at approximately 150 days of growth was purchased from local suppliers. The waste mango and hay were ground in a mixed mill (2–3 cm screen) (M.A.GRO^®^ TR-3500, Mexico), and the silage (50 kg) was made in a 70 × 120 cm propylene bag, 600 caliber. The composition was 86% mango and 14% pangola grass hay. The remaining air was extracted with a vacuum cleaner (Koblenz^®^, Spain) and the bags were sealed with a Smith tie using commercial raffia. The silage was stored in a galley with an average ambient temperature of 28°C.

### Treatments

The treatments used ingredients from the region (Table-1) and increased the levels of waste mango silage to replace the pangola grass hay and ground corn grain, in accordance with calf requirements [8]. The experimental treatments consisted of 0 (T0), 30 (T1), 45 (T2), and 60% (T3) waste mango silage (Table-2).

**Table-1:** Ingredients used in the preparation of treatments.

Parameters	Waste mango silage	Pangola grass hay	Ground corn	Soybean paste	Sodium bicarbonate	Mineral salt
DM (g/kg)	337	960	921	939	998	976
60°C DM (g/kg)						
OM	937.1	926	914	836.4	nd^a^	nd
CP	68.6	48.1	60.7	494.2	nd	nd
NDF	518.9	778	181	290.3	nd	nd
ADF	306.8	432	58.9	61.6	nd	nd
Hemicellulose	212.1	346	122	228.7	nd	nd
Ash	62.9	74	86.4	163.6	629.2	921.9
Production of biogas *in vitro* (mL/g DM)						
Partial biogas 0–24 h	117.3	87.1	179.0	149.8	nd	nd
Partial biogas 24–48 h	40.0	38.0	47.9	28.2	nd	nd
Partial biogas 48–72 h	29.6	28.7	30.0	27.6	nd	nd
Biogas accumulated at 72 h	187.0	153.9	256.8	205.7	nd	nd

a nd=Not determined, DM=Dry matter, OM=Organic matter, CP=Crude protein, NDF=Neutral detergent fiber, ADF=Acid detergent fiber

**Table-2:** Composition of treatments.

Parameters	T0	T1	T2	T3
Wet base ingredient (g/kg)				
Ground corn kernels	50	38	30	22
Mango waste silage	0	30	45	60
Pangola grass hay	32	14	7	0
Soybean paste	14	14	14	14
Sodium bicarbonate	2	2	2	2
Mineral salt*	2	2	2	2
Chemical composition (g/kg DM)				
DM	954	792	713	616
OM	933	919	927	892
CP	129	136	133	141
NDF	393	330	393	276
ADF	186	173	196	116
Hemicellulose	207	156	196	160
Ash	66	80	72	107

T0=Treatment with 0% inclusion of waste mango silage; T1=Treatment with 30% inclusion of waste mango silage; T2=Treatment with 45% inclusion of waste mango silage; T3=Treatment with 60% inclusion of waste mango silage.

* Campisal^®^, 17.58% calcium, 2.40% phosphorus, 36.50% sodium chloride, 11.70% sulfur, 0.71% zinc, 0.14% copper, 0.0007% iodine, 0.0016% cobalt, 0.0029% selenium, 0.024% fluoride, and 5.0% fluorine. DM=Dry matter, OM=Organic matter, CP=Crude protein, NDF=Neutral detergent fiber, ADF=Acid detergent fiber

### *In vitro* test

The culture medium was prepared according to the method outlined by Cañaveral-Martínez *et al*. [9]. In a serological vial (120 mL) 0.5 g samples of the T0, T1, T2, T3, or ingredients with a particle size of 1 mm were added at a constant weight of 40 mL culture medium. All vials were kept under a continuous flow of CO_2_ to maintain anaerobic conditions, sealed with a neoprene stopper (20 mm Ø) and an aluminum ring with a removable center. Each vial was considered to be a biodigester. At 0 h, the biodigesters were placed in a 39°C water bath and inoculated with 10 mL of fresh rumen fluid (pH = 6.4 and 4.9 × 10^9^ bacterial cells/mL).

Biogas production was measured by the displacement of the plunger of a glass syringe (50 mL; BD Yale^®^, Brazil) at 2, 4, 6, 8, 10, 12, 24, 48, and 72 h of incubation [10]. Partial biogas production was reported at 24, 48, and 72 h, and cumulative biogas production at 72 h. The Gompertz model was used to estimate gas production kinetics [11]. Estimators A, b, and k were estimated by non-linear regression analysis, using the procedure general linear model (GLM) function of the SAS software 9.4 [12]. Methane (CH_4_) production was measured according to the procedure of Torres-Salado *et al*. [13]. CH_4_ production was measured as the displaced NaOH (2N) solution in mL at 24, 48, and 72 h of incubation.

At the end of incubation, the ammonia nitrogen concentration was determined according to the procedure used by McCullough [14], pH of the medium contained in the biodigesters, *in vitro* DM degradation (DMDiv), and percentage degradation of neutral detergent fiber (NDFD) and acid detergent fiber (ADFD) were calculated using the formula described by Hernández-Morales *et al*. [10]. The metabolizable energy (ME) was estimated using the equation reported by Muizzu *et al*. [15].

### *In situ* test

*In situ* digestibility was obtained using two cows with a live weight of 350 ± 30 kg body weight (BW), equipped with a permanent rumen cannula (4’’ internal diameter, Bar Diamond^®^, Parma, Idaho, USA). The cows were housed in individual pens and had free access to 3% of the daily feed, consisting of 50% waste mango silage and 50% commercial concentrate (16% CP; Mirador^®^, Cuajinicuilapa, México) and water.

Samples (5 g) were tested in triplicate, with a 1 mm particle size, for each treatment were placed in poly-silk bags (10 × 20 cm) and sealed with plastic straps (100 × 2.5 mm). The bags were soaked in a 39°C water bath for 10 min and then incubated in a rumen sample of each cow for 3, 6, 9, 12, 24, 48, or 72 h. The average ruminal variables during the trial are shown in Table-3. The bags were attached to a galvanized iron chain (1.5 × 100 cm) and fixed to the rumen cannula plug. The order of introduction into the rumen was inverse to the incubation time. Subsequently, once removed the bags were rinsed with cold running water until the rinse water was clear. The 0 h bags were not incubated in the rumen, but rinsed in the same way as those incubated in the rumen.

**Table-3:** Average ruminal variables of the cows used in the *in situ* test.

Time	pH	Total protozoa (10^6^ cells/mL)	Total bacteria (10^9^ cells/mL)	N-NH_3_ (mg/dL)
0	6.40	3.4	4.9	6.83
3	6.57	3.3	4.8	6.81
6	6.48	3.3	4.9	5.81
9	6.30	3.3	4.9	5.71
12	6.21	3.2	4.7	5.51
24	6.10	3.2	5.1	5.09
72	6.54	3.1	5.5	5.08

The bags with the rumen residual were dried at 55°C for 72 h and weighed to determine the DM digestibility (*in situ* DM degradation [DMDis]) by weight difference. The residues from the bags of each experimental treatment in each cow and each time point were pooled to obtain a composite sample. Ash was determined according to Association of Official Analytical Chemists (AOAC) [16]. Organic matter (OM) content was determined by subtracting ash content from 100. Subsequently, the digestibility of *in situ* OM degradation (OMDis) was determined by weight difference before and after ruminal incubation.

The *in situ* digestibility kinetics (a = fast digestible soluble fraction, b = slow or potentially digestible fraction, a + b = maximum potential digestibility, c = speed at which b is digested, k = rumen output cup) and effective digestibility (ED) of DM and OM were estimated by a non-linear regression procedure in SAS^®^ software [11] using the equation described by McDonald [17].

### *In vivo* experiment

Four calves (commercial crosses) with an initial BW of 200 ± 5 kg were housed in individual pens (2.5 × 2 m), provided with a 90% shade net, feeders, and waterers. At the beginning of the experiment, the animals received prophylactic treatment against parasites (Ivermectin 0.2 mg/kg BW through subcutaneous injection), a spray bath with Bovitraz^®^, and ADE vitamins (10 mL/animal through intramuscular injection). The adaptation period was 10 d by means of a gradual supply of the diet. Water was offered *ad libitum*. The diet was offered as two rations per day at 08:00 and 16:00. The experimental design was a 4 × 4 Latin square, each experimental period consisted of 25 days (10 days of adaptation and 15 days of measurement). At the end of each experimental period, the animals were weighed after 10 h of solid feed fasting to adjust for the ration amounts; 10% more than the observed intake was offered.

Daily intake was measured from days 10–25 of each experimental period, where the amount of offered and rejected feed was weighed. Dry matter intake (DMI) was estimated by the difference between the offered and rejected feed. The organic matter intake (OMI), NDF intake (NDFI), ADF intake (ADFI), and crude protein intake (CPI) was estimated according to the DMI, chemical analysis of the treatments, and rejected feed. Samples of the rejected feed were obtained by collecting them for 15 days in each experimental period for each animal and each treatment. They were homogenized and a composite sample was obtained to determine the chemical analysis.

Fecal samples were collected from days 20–25 of each experimental period, 30 g feces was collected directly from the anus of each calf by rectal stimulation. Samples were dehydrated at 60°C in an oven for 48 h, ground, and processed for the apparent digestibility of nutrients (NDF, ADF, DM, OM, and CP) using acid-insoluble ash as an internal marker [18].

On day 25 of each sampling period, 20 mL of ruminal fluid was extracted using an esophageal probe and filtered through double-layer gauze. The pH was immediately measured (Orion^®^ SA210, USA; calibrated at pH 7 and 4). Cellulase enzyme activity was measured using the reducing sugars method as described by Miller [19]. For the total bacterial count (TBC) and protozoan count (PC), the method described by Espinoza-Sánchez *et al*. [3] was used. The number of cellulolytic bacteria (CB) was calculated using the most probable number technique described by Carbajal-Marquez *et al*. [20]. Ammoniacal nitrogen (N-NH_3_) was determined according to the method described by McCullough [13]. Volatile fatty acids (VFA) were determined by depositing 1 mL of rumen fluid into a microcentrifuge tube (Neptune^®^, Mexico; 2 mL) with 0.25 mL of 25% metaphosphoric acid (4:1 ratio). The tubes were centrifuged at 18,800× *g* for 10 min (Hettich Zentrifugen EBA21, Germany). The supernatant was recovered and 1 mL was injected into a gas chromatograph (PerkinElmer^®^, Clarus 500, Massachusetts, USA) equipped with a flame ionization detector and capillary column (Elite FFAP PerkinElmer^®^, Massachusetts, USA). The oven temperature was 115°C for 0.25 min, 125°C for 0.5 min, and 130°C for 5.25 min; the column temperature was 250°C. Nitrogen was the carrier gas, and air and hydrogen were used to generate the flame. Retention times were 1.3, 1.6, and 2.15 min for acetate, propionate, and butyrate, respectively.

### Chemical analysis

The samples were dehydrated in an oven (Riossa, HCF-41, Mexico) at 60°C for 72 h to determine the DM (method 967.03) according to AOAC [16]. Samples were processed in a Thomas-Wiley Mill (Thomas Scientific, Swedesboro, NJ, USA) with a 1 mm sieve. Crude protein (method 920.105) and ash (method 942.05) were determined following the methods proposed by AOAC [16]. Organic matter was calculated by subtracting the percentage of ash from 100. The method proposed by Van Soest *et al*. [21] was used to determine NDF and ADF using a thermostable amylase, and values were expressed, including the residual ash. Hemicellulose was calculated as the difference between NDF and ADF.

### Statistical analysis

The variables obtained from the *in vitro* tests were analyzed in a completely randomized design with five replicates per treatment. Data were analyzed using the GLM procedure of SAS^®^ software [11]. Means were compared using the Tukey test (p < 0.05). The variables obtained from the *in situ* tests were analyzed using the MIXED procedure of SAS^®^ software [11]. Differences between treatment means were determined by the PDIFF option (p < 0.05) of LSMEANS of SAS^®^ software [11]. For the *in vivo* study, the variables were analyzed using the GLM procedure of SAS^®^ software [11] with a 4 × 4 Latin square design. The mean values were compared using the Tukey test (p < 0.05). The response to the increased waste mango silage content in the treatments was calculated using linear and quadratic orthogonal contrasts between the three experiments.

## Results

### *In vitro* test

Partial (24, 48, and 72 h) and cumulative (72 h) biogas production, ME, pH, *in vitro* acid detergent fiber digestibility (ADFDiv), A, and k decreased linearly (p < 0.05), while the DMDiv had a linear increase (p < 0.05) as the percentage of waste mango silage included in the treatments increased. Partial (24, 48, and 72 h), and cumulative (72 h) CH_4_ production, N-NH_3_, NDFDiv, and b did not present with a linear or quadratic trend (Table-4; p > 0.05).

**Table-4:** *In vitro* test of complete diets for calves with increasing amounts of waste mango silage.

Parameters	T0	T1	T2	T3	SEM	Tukey test	Linear	Quadratic
Production of biogas and methane *in vitro* (mL/g DM)								
Partial biogas 0–24 h	167.0^a^	161.9^a^	148.5^b^	149.6^b^	1.61	<0.0001	<0.0001	0.1389
Partial biogas 24–48 h	36.1^ab^	36.9^a^	33.8^b^	34.2^ab^	0.41	0.0189	0.0145	0.7914
Partial biogas 48–72 h	20.3^b^	23.2^b^	29.2^a^	23.4^b^	0.79	0.0002	0.0089	0.0015
Biogas accumulated at 72 h	223.4^a^	222.0^a^	211.5^b^	207.3^b^	1.54	<0.0001	<0.0001	0.5404
Partial methane 0–24 h	35.8^b^	41.5^a^	36.2^ab^	38.0^ab^	0.77	0.0271	0.8368	0.1752
Partial methane 24–48 h	10.6^a^	9.0^a^	11.2^a^	10.2^a^	0.35	0.1362	0.8028	0.6423
Partial methane 48–72 h	5.2^a^	5.6^a^	3.9^a^	5.2^a^	0.30	0.1682	0.4722	0.4292
Methane accumulated at 72 h	51.7^a^	56.2^a^	51.3^a^	53.4^a^	0.95	0.258	0.9815	0.5353
Metabolizable energy (Mcal/kg DM)	1.78^a^	1.76^a^	1.67^b^	1.69^b^	0.01	<0.0001	<0.0001	0.1142
pH	6.61^ab^	6.62^a^	6.56^bc^	6.56^c^	0.008	0.0047	0.0012	0.741
Ammonia nitrogen (mg/dL)	3.61^a^	4.03^a^	4.16^a^	4.16^a^	0.369	0.7684	0.3574	0.632
Degradation (g/kg)								
DM	746^a^	755^a^	745^a^	768^a^	1.46	0.0586	0.0457	0.2348
NDF	593^a^	542^a^	619^a^	530^a^	1.94	0.0716	0.3045	0.4174
ADF (%)	598^a^	582^ab^	625^a^	480^b^	0.20	0.0108	0.0175	0.0246
A (mL/g MS)	203.6^a^	199.2^a^	187.2^b^	185.4^b^	1.588	<0.0001	<0.0001	0.5417
k (h)	3.149^a^	3.050^ab^	2.892^b^	2.891^b^	0.030	0.0014	0.0002	0.3309
b (mL/h)	0.164	0.172	0.159	0.167	0.002	0.2419	0.8481	0.9009

^a,b,c^Means in a row with different letters are different (p < 0.05). SEM=Standard error of the mean; T0=Treatment with 0% inclusion of waste mango silage; T1=Treatment with 30% inclusion of waste mango silage; T2=Treatment with 45% inclusion of waste mango silage; T3=Treatment with 60% inclusion of waste mango silage; A=Total biogas production potential; k=Lag time; b=Constant rate of biogas production from potentially degradable material, DM=Dry matter, NDF=Neutral detergent fiber, ADF=Acid detergent fiber

### *In situ* test

*In situ* dry matter degradation and OMDis at 3, 6, 9, 12, 24, 48, and 72 h showed a linear increase (p < 0.05). The digestibility kinetics of DMDis showed that a and k did not have a linear or quadratic effect (p > 0.05). However, b, a + b, c, and ED showed a linear increase (p < 0.05). The digestibility kinetics of OMDis showed that a, a + b, c, and ED increased linearly (p < 0.05), while b presented with a quadratic behavior (p < 0.05). In addition, k did not show any orthogonal effect (p > 0.05). This behavior was a function of the content of waste mango silage in the diets (Table-5).

**Table-5:** *In situ* test of complete diets for calves with increasing amounts of waste mango silage.

Parameters	T0	T1	T2	T3	SEM	Tukey test	Linear	Quadratic
DMD (g/kg)								
3 h	376^a^	379^a^	381^a^	444^b^	8.0	0.0027	0.0013	0.0148
6 h	415^a^	444^ab^	478^b^	502^b^	11.4	0.003	0.0004	0.8143
9 h	490^a^	481^a^	486^a^	535^a^	15.6	0.1265	0.08	0.0969
12 h	491^a^	528^a^	526^a^	568^ab^	16.2	0.0633	0.0142	0.8853
24 h	636^a^	648^a^	666^a^	696^ab^	12.4	0.0479	0.0087	0.4943
48 h	740^a^	741^a^	752^a^	785^b^	4.4	0.0003	0.0008	0.0388
72 h	782^a^	738^a^	788^a^	812^a^	10.2	0.0681	0.0894	0.0737
a (%)	67^a^	58^a^	64^a^	68^a^	7.2	0.3732	0.5901	0.1519
b (%)	674^a^	670^a^	678^a^	696^a^	8.6	0.1057	0.0425	0.138
a+b (%)	741^abc^	729^b^	742^abc^	764^c^	4.4	0.0292	0.023	0.0284
c (%/h)	0.115^a^	0.138^ab^	0.128^ab^	0.149^b^	0.01	0.0499	0.0209	0.9461
k (%/h)	0.063^a^	0.066^a^	0.064^a^	0.050^a^	0	0.5975	0.3239	0.3799
ED (%)	48.82^a^	50.84^a^	51.48^a^	60.34^a^	1.89	0.1026	0.0308	0.2881
OMD (g/kg)								
3 h	364^a^	337^b^	343^b^	410^c^	7.5	<0.0001	0.0001	<0.0001
6 h	403^a^	431^b^	465^c^	474^c^	11.9	<0.0001	<0.0001	0.0648
9 h	498^a^	461	471^ab^	518^a^	17.5	0.0009	0.0368	0.0002
12 h	485^a^	484^a^	516^b^	576^c^	15.2	<0.0001	<0.0001	0.0004
24 h	639^a^	655^a^	656^a^	719^b^	12.4	<0.0001	<0.0001	0.0003
48 h	747^a^	758^ab^	773^b^	784^b^	3.8	0.0005	<0.0001	0.9262
72 h	789^a^	778^a^	795^ab^	807^b^	4.3	0.0011	0.0009	0.0058
a (%)	248^a^	250^a^	268^b^	268^b^	9.4	0.0001	<0.0001	0.6777
b (%)	566^a^	551^b^	544^c^	559^ab^	6.3	0.0039	0.0737	0.001
a+b (%)	814^a^	802^b^	812^a,b^	828^c^	5.8	0.0007	0.0021	0.0006
c (%/h)	0.059^a^	0.055^a^	0.055^a^	0.077^b^	0.01	<0.0001	<0.0001	<0.0001
k (%)	0.041^a^	0.043^a^	0.042^a^	0.041^a^	0.0002	0.1106	0.5445	0.0319
ED (%)	54.80^a^	55.91^a,b^	57.05^b^	61.37^c^	0.94	<0.0001	<0.0001	0.0007

^a,b,c^Means in a row with different letters are different (p < 0.05). SEM=Standard error of the mean, T0=Treatment with 0% inclusion of waste mango silage, T1=Treatment with 30% inclusion of waste mango silage, T2=Treatment with 45% inclusion of waste mango silage, T3=Treatment with 60% inclusion of waste mango silage, a=Fast digestible soluble fraction, b=Slow or potentially digestible fraction, a+b = Maximum potential digestibility, c=Speed at which b is digested, k=Rumen output cup, ED=Effective degradability, DMD=Dry matter digestibility, OMD=OM digestibility

### *In vivo* experiment

Dry matter, OM, and CP intake increased linearly (p < 0.05) with increased waste mango silage in the diets, while NDFI and ADFI displayed a quadratic trend (p < 0.05), indicating an increase in fiber consumption in T1 and T2 compared to T0, but decreased in fiber consumption in T3 compared to T1 and T2, but showing no difference with T0 (Table-6).

**Table-6:** *In vivo* experiment of complete diets for calves with increasing amounts of waste mango silage.

Parameters	T0	T1	T2	T3	SEM	Tukey test	Linear	Quadratic
Intake (kg/day)								
DM	7.3	8.2	9.2	9.0	0.554	0.066	0.018	0.264
OM	7.0	7.8	8.9	8.3	0.515	0.073	0.032	0.134
CP	0.9^b^	1.2^ab^	1.3^ab^	1.3^a^	0.082	0.031	0.007	0.256
NDF	2.6^b^	2.8^b^	3.7^a^	2.5^b^	0.216	0.005	0.379	0.005
ADF	1.1^b^	1.5^ab^	1.8^a^	1.1^b^	0.12	0.0022	0.536	0.0006
Apparent digestibility (g/kg)								
DM	730	897	747	918	3.55	0.083	0.123	0.97
OM	748	907	770	921	3.45	0.127	0.159	0.937
CP	739	904	796	917	3.25	0.191	0.153	0.721
NDF	670^a^	848^b^	669^a^	867^b^	3.20	0.047	0.11	0.85
ADF	594^a^	826^b^	596^a^	813^b^	3.65	0.026	0.11	0.885
Hemicellulose	738	873	742	906	2.86	0.105	0.139	0.777
Ruminal characteristics								
pH	6.42	6.62	6.57	6.57	0.083	0.798	0.563	0.538
Total protozoa (10^6^ cells/mL)	3.8	3.0	3.8	3.8	0.066	0.888	0.846	0.649
Total bacteria (10^9^ cells/mL)	4.9	5.5	5.1	4.6	0.319	0.836	0.73	0.45
CB (10^7^ cells/mL)	4.5	0.6	3.5	3.4	1.02	0.768	0.96	0.513
N-NH3 (mg/dL)	6.93	5.08	3.81	5.09	0.683	0.436	0.271	0.259
Cellulase (mU/mL)	11.14	12.09	12.94	15.1	0.675	0.193	0.048	0.617
VFA (mmol/L)	41.79	42.77	42.58	41.66	0.997	0.977	0.832	3.596
Acetate (mmol/L)	29.55	30.11	30.30	26.15	0.581	0.370	0.347	2.065
Propionate (mmol/L)	6.74	6.60	6.14	6.45	0.976	0.774	0.832	0.528
Butyrate (mmol/L)	5.50	6.06	6.14	9.07	0.558	0.241	0.544	1.356
Acetate/propionate ratio	4.4	4.8	4.8	4.2	0.741	0.794	0.324	0.184

^a,b,c^Means in a row with different letters are different (p < 0.05). SEM=Standard error of the mean; T0=Treatment with 0% inclusion of waste mango silage; T1=Treatment with 30% inclusion of waste mango silage; T2=Treatment with 45% inclusion of waste mango silage; T3=Treatment with 60% inclusion of waste mango silage, DM=Dry matter, OM=Organic matter, CP=Crude protein, NDF=Neutral detergent fiber, ADF=Acid detergent fiber

The apparent digestibility of DM, OM, CP, and hemicellulose showed neither a linear nor quadratic orthogonal effect, nor were the treatments statistically different (p > 0.05). The NDFD and ADFD in the *in vivo* experiment presented with a quadratic behavior (p < 0.05), where the digestibility of T1 NDF and ADF increased with respect to T0; but T2 showed a reduction and T3 an increase (Table-6).

The ruminal variables of pH, N-NH_3_, TBC, acetate, propionate, butyrate, VFA, acetate: propionate ratio, CB, and PC did not show a linear or quadratic effect (p > 0.05). However, cellulase enzyme activity showed a linear increase (p < 0.05; Table-6).

## Discussion

### *In vitro* test

Biogas production results from the fermentation of soluble sugars, cell wall polysaccharides, and lignin-bound carbohydrates [22]. The biogas production decreased (Table-4) as the percentage of waste mango silage inclusion in the treatment diets increased can be explained by lower availability of readily fermentable carbohydrates associated with the diets (Table-2) as there was a reduced availability of corn [7] as the waste mango silage content increased. The cumulative biogas production in this study (Table-4) was 28.12% higher than that reported in calf diets containing 49% ground corn, 21% pangola grass hay, 5% sugarcane molasses, 11% soybean paste, 3% mineral mix, and 1% urea [23].

In the first 24 h, the average partial CH_4_ production of the treatments containing waste mango silage (T1, T2, and T3) was 3.7% higher than in T0. At 48 h, T2 and T3 produced 1.9% more CH_4_ than T0. However, at 72 h these treatments produced 8.06% less CH_4_ than T0. The average cumulative CH_4_ production of the treatments represented 24.63% of the biogas produced (Table-4). This production is due to the soluble carbohydrate content available for fermentation that produces VFA, CO_2_, and H_2_ [24] and the fermentation of structural carbohydrates where acetic acid, CO_2_, and H_2_ are produced [25]. Therefore, CO_2_ and H_2_ are substrates for the metabolism of methanogenic archaea, where CH_4_ is the final product [26]. Lower values of cumulative CH_4_ production were reported in calf diets with 50% ground corn, 28% pangola grass hay, 5% cane molasses, 13% soybean meal, 3% mineral mix, and 1% urea (51.73 mL/g DM) [23].

ME decreased as mango silage increased in the treatments (Table-4) due to the decrease in available starch in the treatments (Table-2). Espinoza-Sánchez *et al*. [3] reported a 1.7 Mcal/kg DM of ME in diets of lambs containing 40% mango, 25% *Samanea saman* pods, 6% molasses, 26% pangola grass hay, 2% minerals, and 1% urea, a similar value to the average energy content of the diets evaluated in the treatments of this study. The pH of the culture medium (Table-4) of the evaluated treatments did not interfere with the growth of CB and their enzymatic activity, given that a pH close to neutral was maintained [27]. The N-NH_3_ concentrations in the mediums depended on the degradability of the nitrogenous fraction [28], and in our study, an average concentration of 3.99 mg N-NH_3_/dL was recorded in the evaluated treatments (Table-4), a concentration that is lower than that required for a maximum DMDis rate (20–27 mg/dL) [29].

*In vitro* DM degradation increased as the amount of pangola hay in the treatments decreased and mango silage increased (Table-4). The values of DMDiv and NDFDiv (Table-4) suggest that these are treatments with a low fiber concentration (Table-2) and that nutrient intake, energy content, and DMI are not compromised when it is offered to the animal [30]. The decrease in detergent fiber degradation is related to the reduced starch availability when the amount of corn is reduced, and mango silage is increased. The values of detergent fiber degradation in the treatments (Table-4) can be attributed to the fact that the hay used in making the waste mango silage received a chemical treatment with lactic acid to improve the availability of structural carbohydrates attached to lignin [31].

Espinoza-Sánchez *et al*. [3] reported 743 g/kg DMDiv and 579 g/kg NDFDiv in the diets for lambs containing 40% mango silage, values which are similar to the treatments used in this study (Table-4). However, these values are lower than those reported in calf diets (815 g/kg DMDiv and 647 g/kg NDFDiv) with 49% ground corn and 21% pangola grass hay [23] under *in vitro* conditions.

Variables of *in vitro* gas fermentation kinetics constitute a new approach to evaluating feeds and diets [32] or to interpreting the growth curve of the inoculum. The variables A and k decreased as the amount of waste mango silage increased. The average value of b was 0.165 mL/h (Table-4), indicating a higher fermentation rate compared to that reported by López-Vigoa *et al*. [33], who reported 0.032 mL/h for b, 178.7 mL/g DM for A, and 2.45 h for k, in supplements with 20% sugarcane, 70% Guinea grass, and 10% Leucaena (Table-4).

### *In situ* test

*In situ* DM degradation and OMDis increased as a function of waste mango silage in the treatments (Table-5), indicating that the use of silage improved the chemical composition and digestibility of diets because of lactic fermentation [34]. The production of lactic acid increased the permeability and solubilization of the lignin contained in the waste mango silage forage by hydrolyzing the phenolic complexes, which increased microbial adhesion to the feed particles in the waste mango silage treatments [35].

The treatments in our study averaged 780 g/kg DMDis at 72 h (Table-5), which is a higher value than that found in the *in vitro* study (Table-4). However, these values are lower than those published by Medina-Romo *et al*. [36], who reported 871 g/kg DMDis in a cattle diet containing 20% corn stover, 20% alfalfa hay, 26.7% commercial concentrate (18% CP), and 33.3% cactus meal. The discrepancies between this study and those reported by the aforementioned authors are related to the composition and efficiency of the rumen microbiota present in the cattle used for the *in situ* test [37] and the feed offered during the test [35]. This is because in our study, the diet was based on 50% mango silage and 50% commercial feed (12% CP), and the previous studies [36, 38] used to compare results fed the cattle with the same diet they evaluated.

The *in situ* digestibility kinetics reflect the ruminal digestibility pressure integrated by the microorganism-enzyme-substrate interaction [35]. The average of the DMDis kinetics of this study for a, b, k, and c were 6.48, 67.98, 0.24, and 0.53, respectively (Table-5), which are lower than those reported by Laíño *et al*. [38] for the DMDis kinetics (a = 9.04, b = 57.84, k = 6.70 and c = 33.12) in a diet for fattening cattle. Likewise, the average values of OMDis kinetics (a = 25.91, b = 55.54, k = 0.167, c = 0.06; Table-5) were higher than those published by Laíño *et al*. [38] for the OMDis kinetics (a = 3.86, b = 65.59, k = 6.43 and c = 30.55) of a diet containing 27% ground corn, 30% passion fruit meal, 20% cone dust, 18.4% soybean paste, 1.7% calcium carbonate, 1.4% monocalcium phosphate, and 1.5% common salt.

### Performance of the calves

The use of waste mango silage in the calf diet treatments evaluated did not modify the digestibility and biological value of the feed [39], given the values reported for NDFI and ADFI (Table-6). The NDFDiv values indicated that there was no problem with the consumption of the diets in each treatment because the consumption variables satisfied the hedonic signals and energy requirements related to the homeostatic balance of the animals [40]. The behavior of DMI, OMI, and CPI (Table-6) between the treatments is related to the increased waste mango silage amounts used, given the organoleptic characteristics of waste mango silage (palatability) causing an interaction between the homeostatic-homeorhesis and hedonic mechanisms [40].

In the approach of this study, the required DM and CP consumption was estimated to be 7.3 and 0.87 kg/day, respectively, to achieve a daily weight gain of 1.4 kg [7]. However, due to the type of methodological approach in the disposition of the cattle for the evaluated treatments, the daily weight gain was not determined. However, DMI was 0.4, 12.7, 26.7, and 24.3% higher than the estimated DMI for T0, T1, T2, and T3, respectively, while the CPI was 3.4, 37.93, 49.4, and 49.4% (Table-6) higher than the estimated values for T0, T1, T2, and T3, respectively [7]. The presence of waste mango silage in the treatments influenced palatability and thus, voluntary intake [41]. Palatability is assumed to be reflected in animal intake according to NRC [7] parameters. Internal and external factors did not affect nutrient intake and weight gain because they were controlled by the selection and individual management of the calves used in the experiment [41].

In this study, an average DMI of 8.4 kg/day was recorded, a value similar to that reported in the fattening of Charolais calves (DMI of 8.6 kg/day) with a diet based on 9% hay, 39% cellulolytic fibers, and 52% concentrate [42]. The DMI and nutrient DMI of this study (Table-6) were higher than those reported by Do Prado and Martins [43], who estimated the intake of 5.5 kg/day DM, 0.56 kg/day CP, 5.34 kg/day OM, 3.31 kg/day NDF, and 1. 98 kg/day ADF in confined Nellore calves on a diet containing 58% sorghum silage, 19.73% canola bran, and 20.62% ground corn.

The apparent digestibility data in this study showed that in the *in vivo* assay, higher values were obtained than in the *in vitro* degradation (Table-4) and *in situ* digestibility (Table-5). The treatments in the *in vivo* evaluation showed an average of 823 g/kg DMD, 836 g/kg OMD, 771 g/kg NDFD, and 707 g/kg ADFD, while in the *in vitro* evaluation, the average DMDiv was 753 g/kg, NDFDiv was 570 g/kg, and ADFDiv was 570 g/kg. In the *in situ* test, the average DMDis and OMDis was 780 and 792 g/kg, respectively. This behavior is attributed to the conditions under which each experiment was conducted because the *in vivo* digestibility showed mechanical and enzymatic interactions [41] that increased the digestibility of the treatment diets.

*In vivo* digestibility of DM, OM, and CP (Table-6) was not affected by the addition of waste mango silage to the complete diet that meets the requirements for the calves (Table-2). Thus, up to 60% of waste mango silage can be used as a dietary ingredient. This is because the interaction between the rumen bacteria, among others that are responsible for the enzymatic activity, the rate of passage, and the amount of fiber in the diet was maintained [44].

The quadratic behavior of NDFD and ADFD (Table-6) is directly related to the NDF and ADF content of the treatments (Table-2). T0 and T2 quantified an average of 393 and 191 g/kg for NDF and ADF, respectively, which represented 90 g more NDF and 46 g more ADF than the average of T1 and T3. *In vivo* digestibility of DM, NDF, and ADF in the treatments (Table-6) resulted in values higher than those reported by Seger *et al*. [45] and Liu *et al*. [46]. Seger *et al*. [45] published the digestibility of 775, 696, and 715 g/kg for DM, NDF, and ADF, respectively, from a diet containing 20% corn silage, 52% broken corn, and 18% distillers’ grain. The report by Liu *et al*. [46] indicated a measurement of 579, 632, 653, 653, 476, and 415 g/kg for DMD, OMD, CPD, NDFD, and ADFD, respectively, in a finishing diet for Simmental breed bulls composed of 50% corn silage and 26.6% broken corn.

The average of the ruminal variables in the treatments (Table-6) is in accordance with an ideal rumen ecosystem: pH range 5.5–6.9, TBC 10^10^–10^11^ cells/mL, CB 10^7^–10^8^ cells/mL, PC 10^4^–10^6^ cells/mL, N-NH_3_ content 5–8 mg/dL [41, 47], acetate 60–90 mmol/L, propionate15–30 mmol/L, and butyrate 10–25 mmol/L [42]. The efficiency of microbial digestibility in the rumen depends on the stability of the variables in this medium [41]. The treatments averaged a pH 6.5 and N-NH_3_ 3.99 mg/dL in the *in vitro* study (Table-4); for the *in situ* test, an average pH of 6.4 and N-NH_3_ of 5.95 mg/dL were recorded (Table-3), while in the *in vivo* study the pH was 6.5 and N-NH_3_ was 5.22 mg/dL (Table-6). These values are common when starches are used in a whole grain diet, as shown byCui *et al*. [44] which indicated pH ranges of 5.5–6.9, and 5–25 mg/dL of N-NH_3_. Therefore, stability in the ruminal variables can be inferred with supplementation of up to 60% of waste mango silage in calf diets.

Volatile fatty acid production is a result of rumen microbiota metabolism, which is influenced by diet composition and texture, pH, intake frequency, and enzyme activity and accounts for 50%–70% of digestible energy [41]. The VFA values in this study (Table-6) are lower than those published by Seger *et al*. [45], who reported a total VFA production of 86.81 mmol/L, with 46.29 mmol/L acetate, 25.37 mmol/L propionate, and an acetate: propionate ratio of 2.00 with a diet containing 20% corn silage, 52% broken corn, and 18% distiller’s grain. The results of the ruminal variables in our study were higher than those reported by Carbajal-Márquez *et al*. [20], who reported a pH of 6.89, 2.97 mg/dL N-NH_3_, 3.32 × 10^5^ cells/mL PC, 4.21 × 10^9^ cells/mL TBC, 4.80 × 10^7^ cells/mL CB, 15.63 mU/mg cellulase protein, 35.06 mmol/L VFA, 22.23 mmol/L acetate, 8.02 mmol/L propionate, 4.82 mmol/L butyrate, and an acetate: propionate ratio of 2.76 using protein supplementation containing 28% soybean meal, 4% urea, 7% corn grain, 56% hay, and 5% mineral salt.

## Conclusion

The *in vitro*, *in situ*, and *in vivo* experiment results established that up to 60% waste mango silage can be included in a complete diet for calves of 200 ± 5 kg BW in confinement in tropical areas, satisfying their nutritional requirements and maintaining biological stability. In addition, other studies have suggested that producing silages with 86% waste mango and 14% pangola grass hay can be used in diets for calves in confinement in the tropics as a strategy to utilize harvest and feeding residues in ruminants.

## Authors’ Contributions

URC: Conceptualized and designed the study and collected data. PS: Conceptualization, methodology, data curation, resources, project administration and writing - review and editing. JH: Designed and formulated *in vitro* material in the laboratory and supervised data. DH: Designed and formulated *in situ* material in the laboratory and supervised data. MAA: Designed and formulated *in vivo* material and supervised data. URC and PS: Drafted, edited, and critically revised the manuscript. All authors have read, reviewed, and approved the final manuscript.
